# IUC25511-7 MERIT AWARD: Prostate volume changes during longer fractionated and ultra-hypofractionated MR-guided adaptive radiotherapy leveraging the MOMENTUM registry

**DOI:** 10.1093/oncolo/oyag286.010

**Published:** 2026-08-21

**Authors:** S Cooper, H Akhiat, S Alexander, F Alongi, E Blezer, A Choudhury, J Christodouleas, J Diaz, C Gani, S Hafeez, W Hall, P Jeene, M Luzzara, M Nowee, T Schytte, H Verkooijen, D Vesprini, J Van Der Voort Van Zyp, P Westhoff, A C Tree

**Affiliations:** Royal Marsden NHS Foundation Trust / Institute of Cancer Research, London - United Kingdom; Elekta, Stockholm - Sweden; Royal Marsden NHS Foundation Trust / Institute of Cancer Research, London - United Kingdom; IRCCS Ospedale Sacro Cuore-Don Calabria, Cancer Care Center, Negrar Verona - Italy; University Medical Center Utrecht, Utrecht - Netherlands; Division of Cancer Sciences, University of Manchester / The Christie NHS Foundation Trust, Manchester - United Kingdom; Elekta, Stockholm - Sweden; Royal Marsden NHS Foundation Trust / Institute of Cancer Research, London - United Kingdom; Universitätsklinikum Tübingen, Tübingen - Germany; Royal Marsden NHS Foundation Trust / Institute of Cancer Research, London - United Kingdom; Medical College of Wisconsin / Froedtert Hospital, Milwaukee - United States; Radiotherapiegroep, Deventer/Arnhem - Netherlands; Royal Marsden NHS Foundation Trust / Institute of Cancer Research, London - United Kingdom; Netherlands Cancer Institute - Antoni van Leeuwenhoek, Amsterdam - Netherlands; Odense University Hospital, Odense - Denmark; Netherlands Cancer Institute - Antoni van Leeuwenhoek, Amsterdam - Netherlands; Sunnybrook Health Sciences Centre, Toronto - Canada; University Medical Center Utrecht, Utrecht - Netherlands; Radboud Universitair Medisch Centrum, Nijmegen - Netherlands; Royal Marsden NHS Foundation Trust / Institute of Cancer Research, London - United Kingdom

## Abstract

**Background:**

Prostate volume changes during radiotherapy may compromise target coverage. We quantified longitudinal swelling across longer fractionation (≥15 fractions)(LFRT) and stereotactic body radiotherapy (SBRT) regimes during MR-guided adaptive radiotherapy (MRgART) to identify predictors of clinically significant swelling (CSS, defined as ≥ 15% volume increase).

**Methods:**

This multicentre prospective cohort study analysed patients treated on an MR-Linac between January 2020 and April 2025, enrolled in MOMENTUM [6]. Inclusion mandated daily recontouring/adaptation with ≥3 fractions available. Structure volumes were extracted using PyRadiomics (v3.0.1). A hierarchical fuzzy-matching algorithm (Levenshtein distance) harmonised prostate volume nomenclature across centres. Clinical and demographic variables were collected, and fraction 1 was counted as the baseline volume [7]. The proportion of treatment spent with CSS was normalised to the number of fractions. Baseline prostate volume was dichotomised. Linear mixed-effects models (LMM) quantified continuous volume trajectories by cohort. Multivariable logistic regression tested predictors of CSS as a binary outcome.

**Results:**

From 1907 patients, 222 LFRT and 1390 SBRT patients met eligibility. A total of 10297 fractions were analysed (median 20 for LFRT, 5 for SBRT patients), with median baseline prostate volume of 40.0 cm³ (IQR 28.9-53.6) for LFRT and 47.5 cm³ (IQR 35.8-66.0) for SBRT. Incomplete age data excluded 160 patients from LMM. CSS occurred in 15.2% LFRT and 13.1% SBRT total fractions (Figure 1), but SBRT patients spent a higher proportion of treatment in CSS (Figure 2). In LMM, larger baseline prostate volume was associated with reduced mean swelling in SBRT (β = -0.030% per cm³, p < 0.001) but not LFRT (p  =  0.30). However, logistic regression showed larger baseline volume was protective in both cohorts (LFRT: OR 0.38, 95% CI 0.32-0.46; SBRT: OR 0.80, 95% CI 0.69-0.92). In secondary complete-case analysis including ADT and T-stage data (n  =  116 LFRT, n  =  256 SBRT), no significant association with ADT was observed in either cohort (LFRT p  =  0.65; SBRT p  =  0.37), whilst T2-stage disease was associated with increased swelling in SBRT (β  =  +3.0%, p  =  0.018) compared to T1-stage.

**Conclusions:**

SBRT patients experience a faster onset of prostate swelling compared to LFRT. Smaller baseline volumes and T2-stage were associated with increased swelling risk. Incomplete clinical covariate data limits definitive risk stratification, supporting continued fraction-by-fraction adaptive workflows.

